# Cytomegalovirus Colitis Presenting as a Cecal Pseudotumor Mimicking Colorectal Malignancy in a Patient With Advanced HIV: A Case Report

**DOI:** 10.7759/cureus.111572

**Published:** 2026-06-26

**Authors:** Daniela Osowiecki Feldman, Garrett Teskey, Daniel A Ward, Anne K Stewart

**Affiliations:** 1 Internal Medicine, University of California San Diego School of Medicine, San Diego, USA; 2 Internal Medicine, Scripps Mercy Hospital, San Diego, USA; 3 Pathology and Laboratory Medicine, Scripps Mercy Hospital, San Diego, USA

**Keywords:** cecal mass, cytomegalovirus (cmv), cytomegalovirus colitis, hiv aids, infectious pseudotumor

## Abstract

Cytomegalovirus (CMV) is an opportunistic pathogen that frequently involves the gastrointestinal tract in immunocompromised patients, particularly those with advanced HIV infection. CMV colitis typically presents with nonspecific gastrointestinal symptoms and endoscopic ulcerations. Mass-forming lesions, however, are an exceptionally rare manifestation and may mimic malignancy.

We report a case of a 64-year-old man with untreated HIV/AIDS (CD4+ T-cell count 24 cells/μL, HIV RNA 2,748,289 copies/mL) who was brought to the emergency department by ambulance after a fall, along with progressive encephalopathy, and was found to have a cecal mass on imaging despite the absence of gastrointestinal complaints. Colonoscopy demonstrated multiple friable, fungating, malignant-appearing masses and a mass causing mild obstruction at the junction between the ascending colon and the cecum. Despite initial concern for colorectal adenocarcinoma, histopathology revealed CMV inclusions without evidence of malignancy. The patient underwent a right hemicolectomy prior to definitive diagnosis and was treated with ganciclovir followed by valganciclovir. This case highlights the importance of considering CMV in the differential diagnosis of gastrointestinal mass lesions in severely immunocompromised patients in order to potentially avoid unnecessary surgical intervention.

## Introduction

Cytomegalovirus (CMV) is a well-recognized opportunistic pathogen in immunocompromised individuals, particularly those with advanced HIV infection and transplant recipients [1]. In patients with HIV, CMV end-organ disease most commonly occurs with severe immunosuppression (CD4+ T-cell counts <50 cells/μL) and generally targets the retina, lungs, gastrointestinal tract, or central nervous system [2, 3]. 

Gastrointestinal involvement frequently manifests as CMV colitis, which typically presents with nonspecific symptoms such as diarrhea, abdominal pain, fever, and weight loss, along with endoscopic findings of mucosal ulceration [2, 4]. However, CMV infection can rarely produce mass-forming lesions referred to as pseudotumors that may closely mimic gastrointestinal malignancy on imaging and endoscopy [5-15]. CMV-associated pseudotumors are uncommon inflammatory mass-forming lesions characterized by reactive tissue proliferation rather than true neoplastic transformation. These mass-forming lesions are most commonly reported in patients with advanced HIV/AIDS and profound immunosuppression, typically characterized by markedly reduced CD4+ T-cell counts and high HIV viral loads, both of which are major risk factors for opportunistic infections and atypical disease manifestations. The clinical presentation of CMV-associated pseudotumors is often nonspecific and ranges from an absence of gastrointestinal symptoms, as observed in our patient, to manifestations such as abdominal pain, fever, changes in bowel habits, and weight loss [14]. 

Since the widespread implementation of antiretroviral therapy (ART), the incidence of CMV end-organ disease has declined dramatically, rendering such atypical presentations increasingly uncommon in modern clinical practice [2]. Fewer than 25 cases of CMV-associated gastrointestinal pseudotumors have been reported in the literature, highlighting the exceptional rarity of this entity. Despite the widespread use of ART, CMV remains a significant opportunistic pathogen in patients with advanced, untreated HIV and profound immunosuppression. Consequently, rare manifestations such as pseudotumor formation remain clinically challenging and often pose a diagnostic dilemma due to their striking resemblance to gastrointestinal malignancy.

The diagnosis of a CMV-associated pseudotumor relies on histopathologic confirmation, including the identification of characteristic CMV cytopathic changes with intranuclear inclusions and positive CMV immunohistochemical staining. Equally important, alternative etiologies, including malignancy and other infectious or inflammatory processes, must be excluded, making this diagnosis largely one of exclusion.

Given the rarity of CMV-associated pseudotumors, evidence-based management strategies remain poorly defined. Additionally, the diagnostic uncertainty surrounding these lesions may result in invasive interventions, as their clinical, radiographic, and endoscopic appearance often closely resembles that of a neoplastic process.

## Case presentation

A 64-year-old male patient with a history of HIV/AIDS diagnosed 32 years prior, not currently on ART, was brought to the emergency department by ambulance after a fall, along with progressive cognitive decline over several months. His medical history was notable for prior opportunistic infections, including disseminated *Mycobacterium avium *complex, *Pneumocystis jirovecii* pneumonia, and HIV-associated encephalopathy. 

On presentation, the patient denied any gastrointestinal complaints, including abdominal pain, significant changes in bowel habits, melena, or hematochezia. Vital signs revealed a temperature of 38.5°C, heart rate of 142 beats/minute, respiratory rate of 28 breaths/minute, and blood pressure of 124/62 mmHg. He appeared cachectic and chronically ill, with altered mental status but no focal neurologic deficits, and his abdominal examination was benign. 

Laboratory evaluation demonstrated a CD4+ T-cell count of 24 cells/μL and an HIV viral load of 2,748,289 copies/mL. Venous blood gas demonstrated an elevated lactate of 2.8 mmol/L. However, the complete blood count showed no leukocytosis, a hemoglobin of 11.6 g/dL, and a hematocrit of 34.1% (Table 1). 

**Table 1:** Laboratory findings on presentation

Laboratory Test	Findings	Reference Range
CD4 Count	24 cells/µL	430–1800 cells/µL
HIV RNA	2,748,289 copies/mL	<0 copies/mL
Lactate	2.8 mmol/L	0.07–2.10 mmol/L
White Blood Cell Count (WBC)	3.7 K/µL	3.4–11.0 K/µL
Hemoglobin	11.6 g/dL	13.0–17.1 g/dL
Hematocrit	34.10%	39.8–51.5%

Computed tomography (CT) of the chest, abdomen, and pelvis without contrast revealed concentric mass-like thickening of the cecum concerning for neoplasm (Figure 1), along with pulmonary nodular opacities. Of note, typhlitis was not observed despite mass-like thickening of the cecum. Colonoscopy demonstrated multiple friable, ulcerated, fungating masses up to 5 cm in size occupying approximately half of the cecal circumference (Figure 2). Additionally, a partially obstructing mass was noted at the ascending colon-cecal junction, causing mild narrowing with minimal resistance to colonoscope passage, though its size and extent raised concern for potential progression to bowel obstruction.

**Figure 1:**
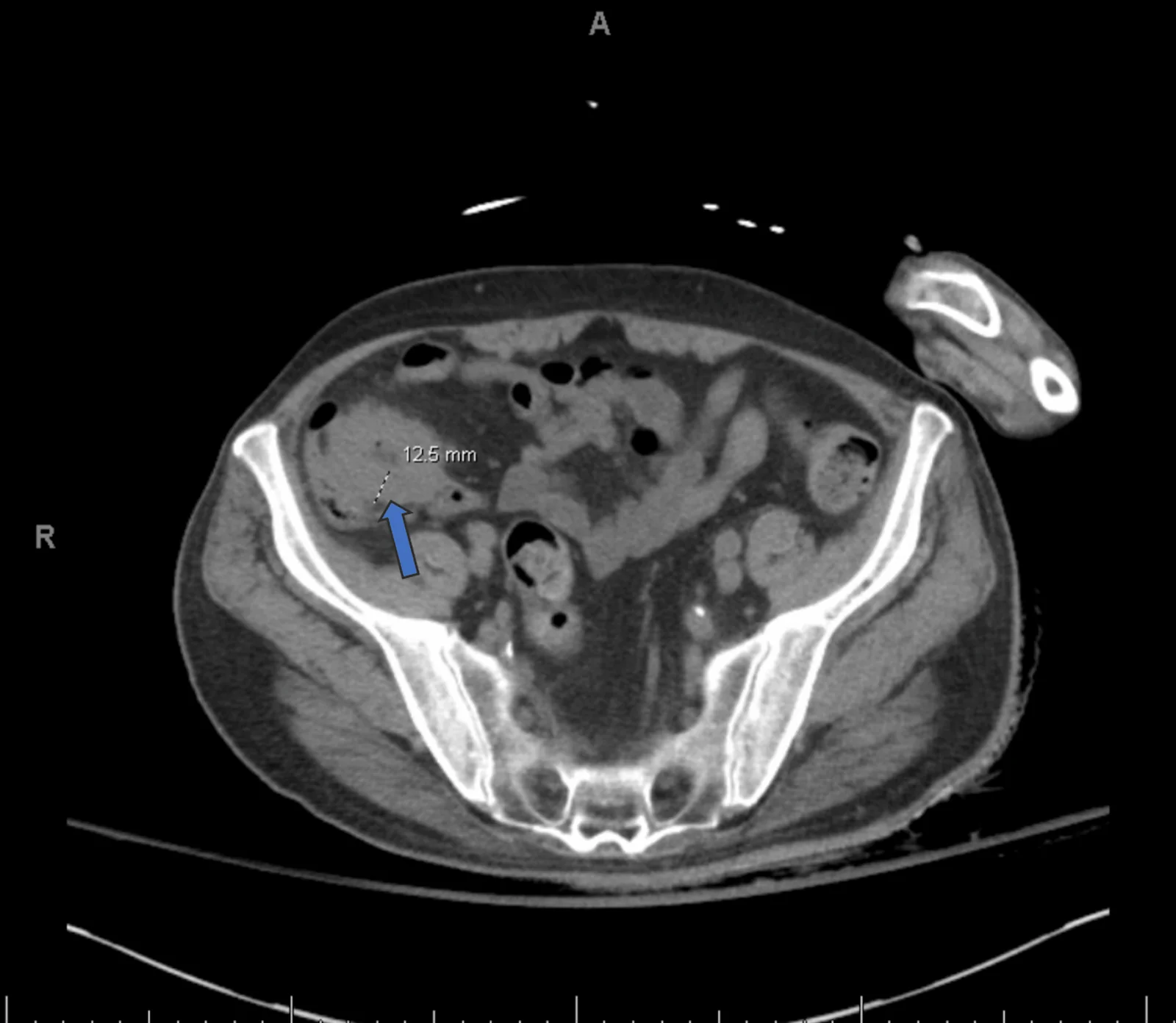
Computed tomography (CT) of the chest, abdomen, and pelvis without contrast demonstrating concentric, mass-like thickening of the cecum (arrow), raising concern for an underlying neoplastic process. Note the loss of normal haustral folds and soft tissue density.

**Figure 2:**
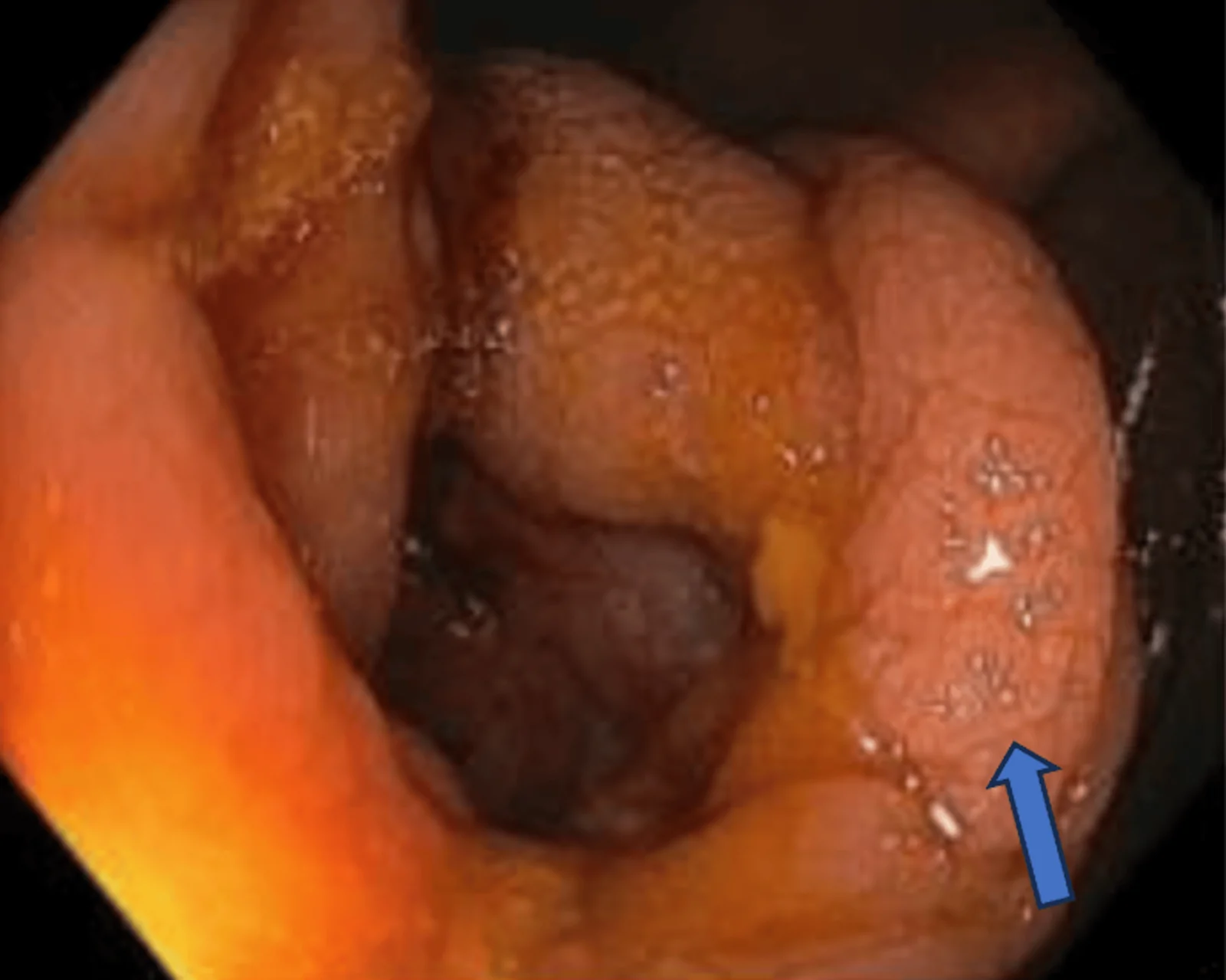
Colonoscopic view of the cecum demonstrating friable mass with irregular borders (arrow), highly suspicious for malignancy.

Initial biopsies revealed inflamed granulation tissue with focal necrosis and CMV-positive cells on immunostaining, without evidence of malignancy (Figures 3-4). Although endoscopic biopsies demonstrated CMV infection without evidence of malignancy, the patient underwent surgical resection due to extensive inflammatory and ulcerative involvement of the cecum, partial luminal compromise from the mass lesion, and persistent concern for an underlying neoplastic process that could not be definitively excluded. Induction therapy with IV ganciclovir was initiated on the same day as resection in the setting of confirmed tissue-invasive CMV infection. Surgical pathology demonstrated transmural lymphoplasmacytic inflammation, ulceration, multinucleated giant cells, granulomas, and CMV inclusions (Figure 5). Five lymph nodes were noted to have multiple granulomas, and the biopsies were again negative for malignancy. Acid-fast staining and fungal studies were negative as well. 

**Figure 3:**
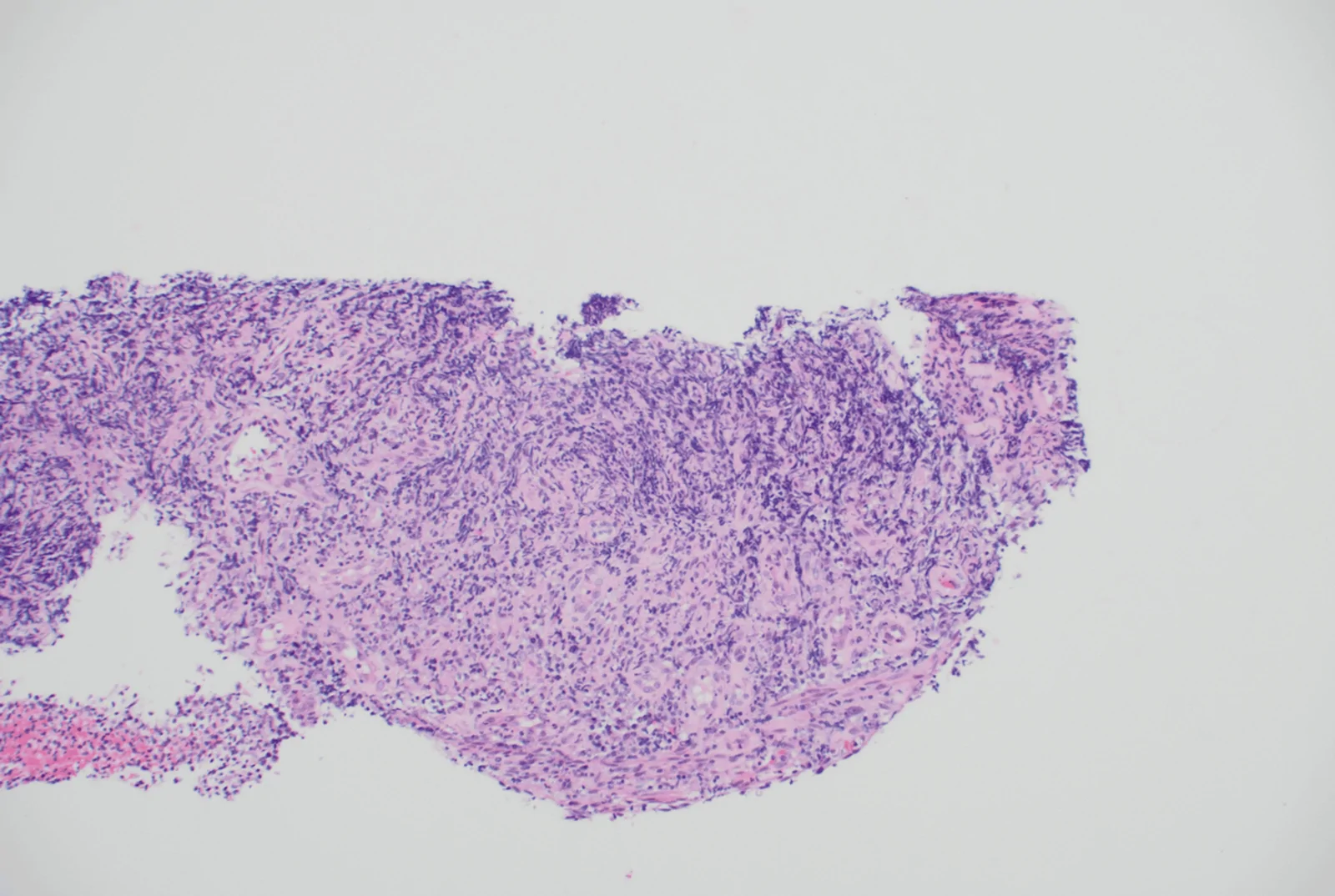
Hematoxylin and eosin (H&E) stain of the cecal biopsy showing ulcerated colonic mucosa with mixed inflammatory infiltrate (10× magnification). Biopsy demonstrating endothelial cells with large ovoid nuclei with basophilic intranuclear inclusions in owl's eye configuration.

**Figure 4:**
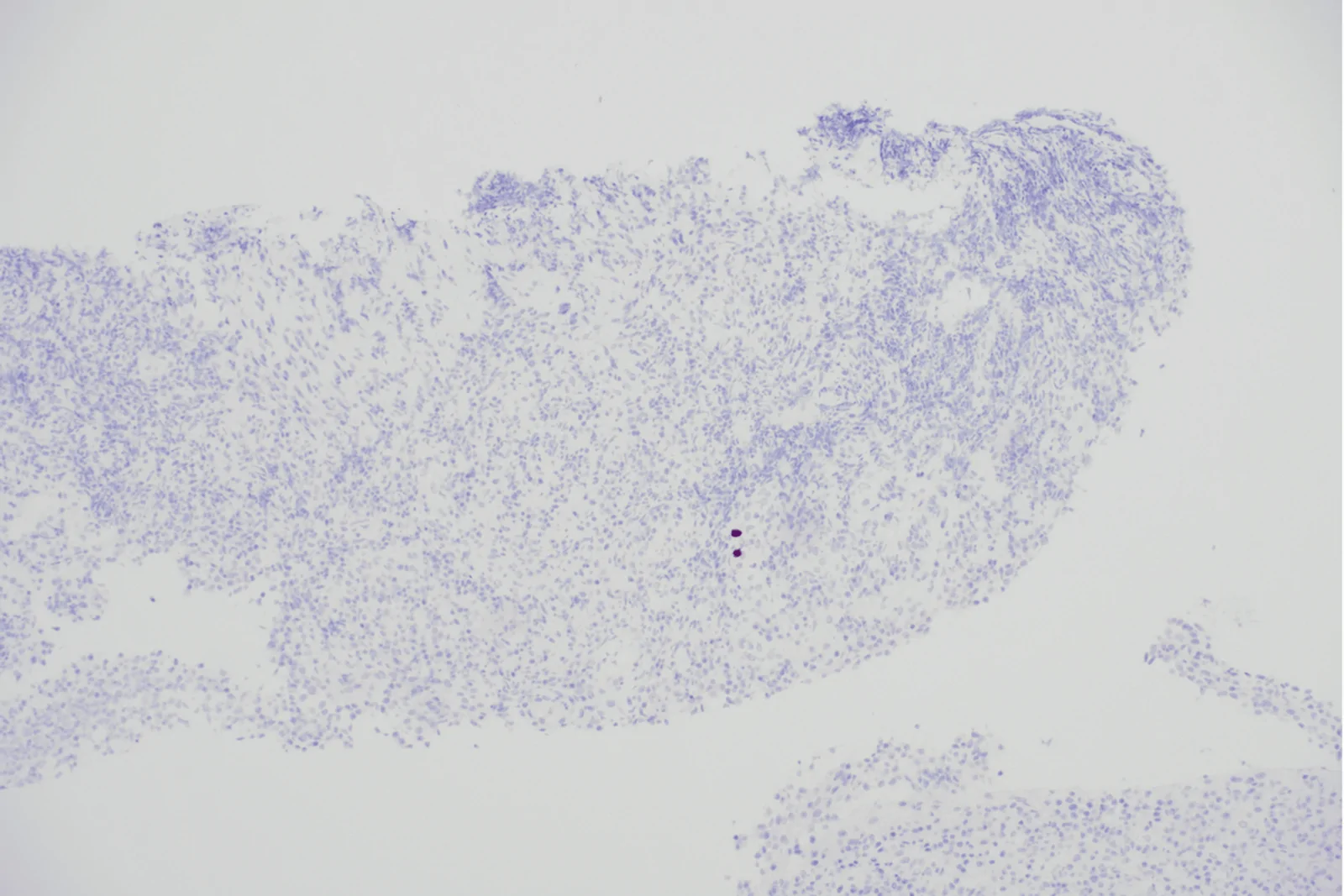
Immunohistochemical stain for cytomegalovirus demonstrating scattered positively stained cells.

**Figure 5:**
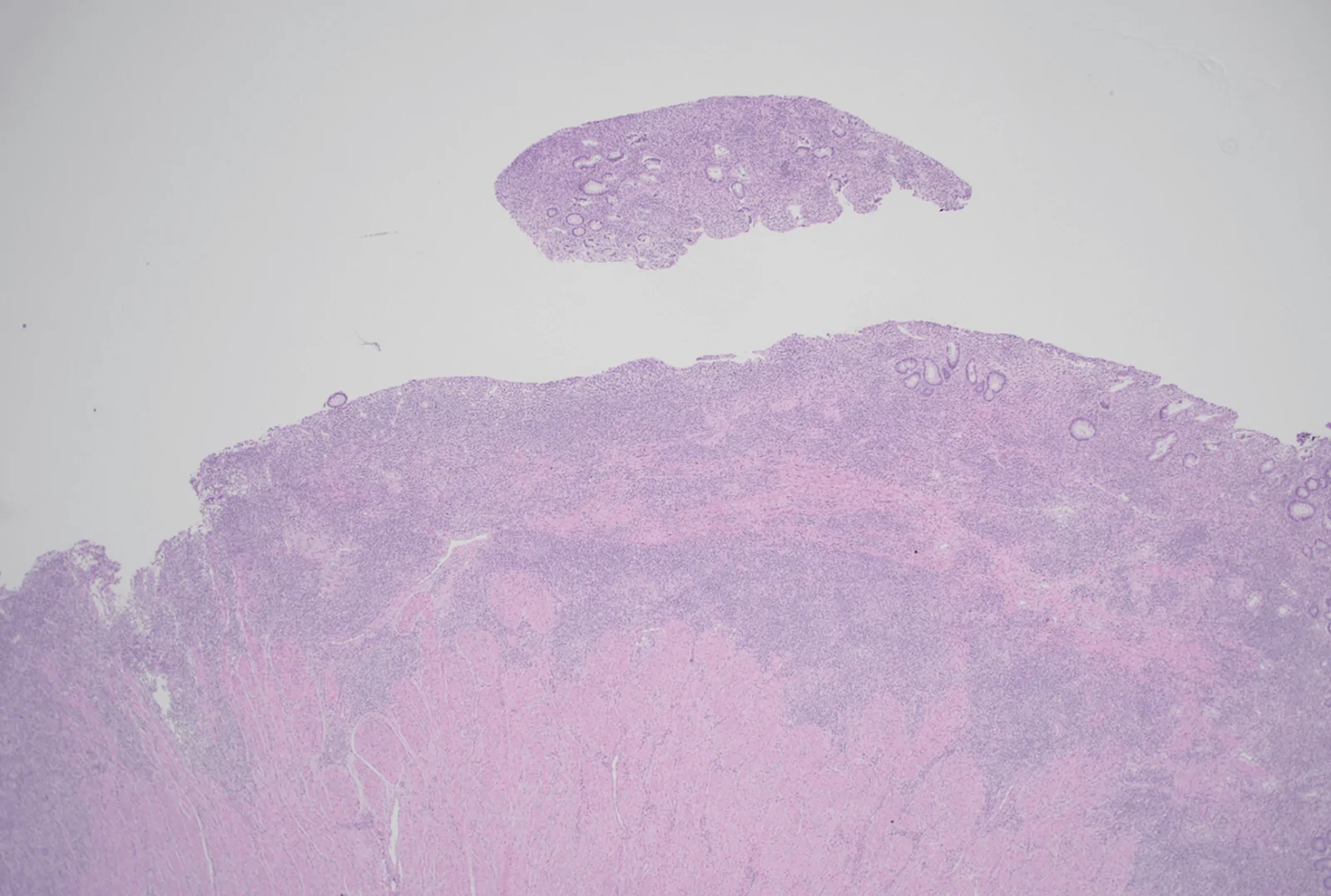
Hematoxylin and eosin (H&E) stain of the resected colonic tissue demonstrating transmural lymphoplasmacytic inflammation, ulceration, and multinucleated giant cells, consistent with cytomegalovirus-associated colitis (4× magnification).

Further evaluation for encephalopathy included magnetic resonance imaging (MRI) of the brain showing confluent white matter changes and cerebrospinal fluid (CSF) analysis demonstrating lymphocytic pleocytosis with elevated protein, consistent with a viral process (Figure 6). CSF studies were negative for CMV PCR and bacterial growth, and additional testing, including Venereal Disease Research Laboratory (VDRL) and fungal cultures and the John Cunningham virus, yielded no detectable abnormalities.

**Figure 6:**
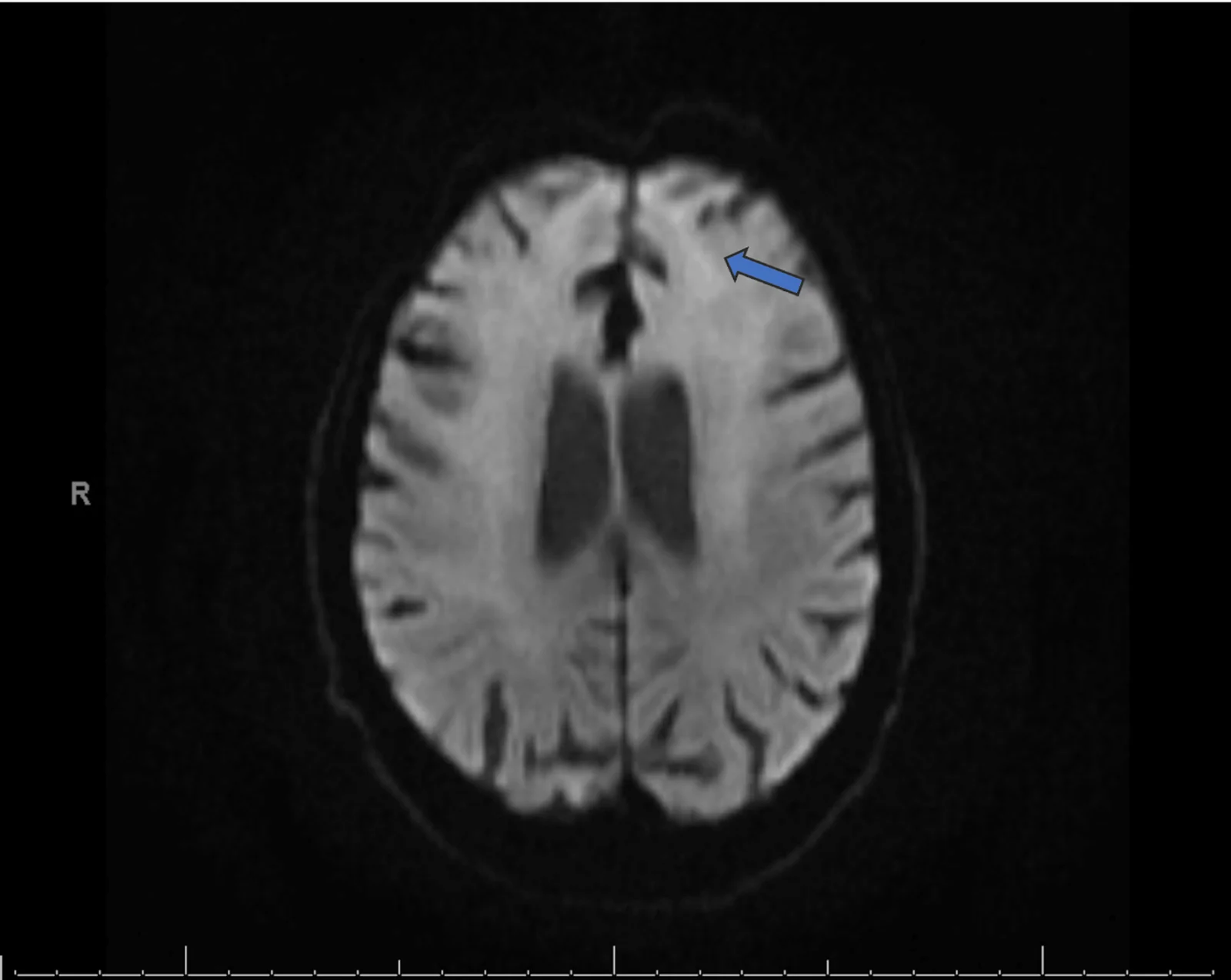
Magnetic resonance imaging (MRI) of the brain demonstrating confluent white matter hyperintensities predominantly involving the frontal lobes (arrow). No acute intracranial abnormalities were identified. Findings are suggestive of HIV-associated encephalopathy in the absence of an alternative explanation.

The patient was ultimately treated with intravenous ganciclovir for 10 days, followed by oral valganciclovir for 21 days. He was also initiated on prophylaxis for opportunistic infections with trimethoprim-sulfamethoxazole and azithromycin. 

## Discussion

Gastrointestinal mass lesions in patients with advanced HIV are most commonly attributed to malignancy, particularly Kaposi sarcoma and non-Hodgkin lymphoma, in the setting of severe immunosuppression with CD4+ T-cell counts <200 cells/μL. Infectious etiologies such as tuberculosis, histoplasmosis, and toxoplasmosis are less common but important considerations [6]. CMV is a rare but recognized cause of pseudotumor formation in the gastrointestinal tract [5-13]. The pathogenesis of CMV-associated pseudotumor formation remains poorly understood, and a definitive mechanism has yet to be established. Current evidence suggests that CMV-induced epithelial and stromal cell proliferation, mediated by viral proteins that disrupt normal apoptotic and cell-cycle regulatory pathways, may contribute to the development of these mass-like lesions. This process is likely exacerbated by chronic infection and impaired immune surveillance in severely immunocompromised hosts [14-16]. These lesions may be indistinguishable from malignancy on imaging and endoscopy, posing a significant diagnostic challenge. In previously reported cases, patients most commonly presented with abdominal pain, fever, or changes in bowel habits [14]. Notably, our patient lacked gastrointestinal symptoms despite extensive colonic involvement, further complicating clinical suspicion. 

A review by Kelesidis et al. identified only 15 reported cases of CMV-associated gastrointestinal pseudotumors in HIV-positive patients from 1980 through 2008 [14]. Including additional cases reported in the literature since then, fewer than 25 cases have been documented overall, underscoring the rarity of this presentation [6-14]. In the Kelesidis et al. review, CMV pseudotumors in HIV-positive patients most commonly involved the colon (6/14 cases, 42.9%), particularly the cecum and adjacent ileum, with other reported sites including the stomach, esophagus, and small bowel [14]. This is consistent with our case, which involved the colon and, in particular, the cecum. Of the 14 cases reviewed by Kelesidis et al., a diagnostic surgical approach was performed in eight of the 14 cases. In regard to treatment, five patients were treated medically with ganciclovir, two of the cases were treated with both surgery and ganciclovir, and two additional cases were initially treated with ganciclovir but changed to foscarnet due to side effects. No information with regards to the medical treatment was provided for the remainder of the reviewed cases. Notably, they described the first reported case of a CMV-associated pseudotumor in a patient with AIDS that resolved completely with oral valganciclovir therapy alone [14]. However, the extent to which antiviral therapy alone is sufficient to achieve complete resolution of CMV-associated pseudotumors remains unclear. Consequently, in our case, it is uncertain whether the lesion would have regressed with antiviral treatment alone or whether surgical resection would ultimately have been required. =

This case illustrates several important clinical considerations. First, CMV should remain in the differential diagnosis of mass-like gastrointestinal lesions in severely immunocompromised patients, particularly those not receiving ART. Second, histopathologic confirmation is critical, as endoscopic and radiographic findings alone may be misleading. Finally, early recognition of CMV pseudotumor may allow for medical management with antiviral therapy and potentially avoid unnecessary surgical intervention. Although data are limited, prior reports suggest that CMV pseudotumors may resolve with antiviral therapy alone [14]. In this case, the combination of extensive inflammatory and ulcerative involvement of the cecum, partial luminal compromise, and persistent concern for an underlying malignancy resulted in significant diagnostic uncertainty and ultimately prompted surgical resection.

## Conclusions

CMV colitis can rarely present as a mass-forming lesion that mimics gastrointestinal malignancy in patients with advanced HIV. Awareness of this entity is essential, as misdiagnosis may lead to invasive surgical interventions and delayed antiviral therapy. Importantly, CMV pseudotumors may occur even in the absence of gastrointestinal symptoms, further underscoring the need for a high index of suspicion in this population. In immunocompromised patients presenting with gastrointestinal mass lesions, consideration of infectious etiologies and prompt histopathologic confirmation are essential to avoid misdiagnosis and guide appropriate management.
